# Adenosine Deaminase Acting on RNA-1 (ADAR1) Inhibits HIV-1 Replication in Human Alveolar Macrophages

**DOI:** 10.1371/journal.pone.0108476

**Published:** 2014-10-01

**Authors:** Michael D. Weiden, Satomi Hoshino, David N. Levy, Yonghua Li, Rajnish Kumar, Sean A. Burke, Rodney Dawson, Catarina E. Hioe, William Borkowsky, William N. Rom, Yoshihiko Hoshino

**Affiliations:** 1 Division of Pulmonary & Critical Care Medicine, Departments of Medicine and Environmental Medicine, New York University School of Medicine, New York, New York, United States of America; 2 Department of Pathology, New York University School of Medicine, New York, New York, United States of America; 3 Department of Pediatrics, New York University School of Medicine, New York, New York, United States of America; 4 Department of Basic Sciences and Craniofacial Biology, New York University College of Dentistry, New York, New York, United States of America; 5 VA New York Harbor Healthcare System–Manhattan Campus, New York, New York, United States of America; 6 Lung Institute, Department of Medicine, University of Cape Town, Cape Town, South Africa; University of Medicine and Dentistry of New Jersey - New Jersey Medical School, United States of America

## Abstract

While exploring the effects of aerosol IFN-γ treatment in HIV-1/tuberculosis co-infected patients, we observed A to G mutations in HIV-1 envelope sequences derived from bronchoalveolar lavage (BAL) of aerosol IFN-γ-treated patients and induction of adenosine deaminase acting on RNA 1 (ADAR1) in the BAL cells. IFN-γ induced ADAR1 expression in monocyte-derived macrophages (MDM) but not T cells. ADAR1 siRNA knockdown induced HIV-1 expression in BAL cells of four HIV-1 infected patients on antiretroviral therapy. Similar results were obtained in MDM that were HIV-1 infected *in* *vitro*. Over-expression of ADAR1 in transformed macrophages inhibited HIV-1 viral replication but not viral transcription measured by nuclear run-on, suggesting that ADAR1 acts post-transcriptionally. The A to G hyper-mutation pattern observed in ADAR1 over-expressing cells *in* *vitro* was similar to that found in the lungs of HIV-1 infected patients treated with aerosol IFN-γ suggesting the model accurately represented alveolar macrophages. Together, these results indicate that ADAR1 restricts HIV-1 replication post-transcriptionally in macrophages harboring HIV-1 provirus. ADAR1 may therefore contribute to viral latency in macrophages.

## Introduction

*Mycobacterium tuberculosis* infects one-third of the world’s human population. In 2012, approximately 8.6 million people developed active TB with 1.3 million deaths of whom 320,000 were HIV/TB co-infected [1], There is a mutual interaction between HIV-1 and tuberculosis, each aggravating the other by reducing CD4+ cells and enhancing inflammation and HIV-1 replication [2], [3]. Interferons (IFN) are secreted proteins central to both innate and cellular immunity. The IFN-γ pathway is critical to host defense in *M. tuberculosis*-exposed mice [4], [5]. In humans, mutational defects in the IFN-γ receptor or signaling intermediates result in disseminated mycobacterium infection [6]. IFN-γ is also antiviral, reducing HIV-1 replication in macrophages [7], [8]. In 2001, IFN-γ was used subcutaneously to prevent opportunistic infections (OIs) in HIV-1-infected patients but failed to reach significance due to over dispersion in the model (1.71 OIs for IFN-γ recipients versus 3.45 OIs for controls over 48 months, NS), and had reversible adverse events [9].

One of the interferon-mediated mechanisms restricting viral replication is induction of RNA editing enzymes leading to hyper-editing and inactivation of viral RNAs in avian leucosis virus [10], measles virus [11], parainfluenza virus [12], polyoma virus [13], and respiratory syncytial virus [14]. Adenosine deaminase acting on RNA 1 (ADAR1) is one of those RNA editing enzymes. It catalyzes the deamination of adenosine (A) to inosine (I) that is then recognized as guanosine (G) during subsequent rounds of replication [15]. ADAR1 exists as two isoforms: a 150-kDa form whose expression is induced by IFN, and a constitutively expressed 110-kDa form. The 150-kDa form is the only ADAR family member expressed in the cell cytoplasm suggesting a unique role for this IFN-induced RNA editing enzyme [16]. Recently, RNA editing by the 150-kDa isoform of ADAR1 was shown to be a potent inhibitor of HIV-1 replication [17] raising the possibility that ADAR1 also acts on HIV-1 *in* *vivo*.

ADAR1 is similar to the RNA editing enzyme cytidine deaminase APOBEC3G, shown to be an innate immune inhibitor of HIV-1 replication in lymphocytes [18]. APOBEC3G produces a cytidine to uridine mutation in the minus strand DNA during reverse transcription. It therefore effectively prevents proviral integration in T cells. Unlike APOBEC3G, ADAR1 is not effective in inhibiting HIV-1 replication in lymphocytes [19].

HIV-1-infected individuals who remain asymptomatic despite prolonged infection are designated as long-term non-progressors (LTNP). These individuals frequently have vigorous anti-HIV-1 immune responses detectable with the IFN-γElispot assay or intracellular IFN-γ staining [20], [21]. When the HIV-1 sequences of LTNP were examined, A-to-G and G-to-A hyper-mutations were found throughout the entire genome, suggesting a role for RNA editing enzymes in viral replication [22], [23].

During pathophysiological investigation of patient-derived material from the lungs of HIV/TB co-infect patients treated with aerosolized IFN-γ, we observed hyper-mutation of lung derived HIV-1 and induction of ADAR1 mRNA in bronchoalveolar lavage cells retrieved from these patients. We therefore developed cell culture models to test if ADAR1 inhibited HIV-1 replication and produced A to G mutations in macrophages. We chose to use macrophage models because alveolar macrophages are a major source of HIV-1 replication during tuberculosis and because these long lived cells are a potential reservoir for latent virus during prolonged antiretroviral therapy [24].

## Results

### The HIV-1 envelope glycoprotein V3 region in bronchoalveolar lavage (BAL) fluids is highly mutated when patients are exposed to IFN-γ

**Figure 1:**
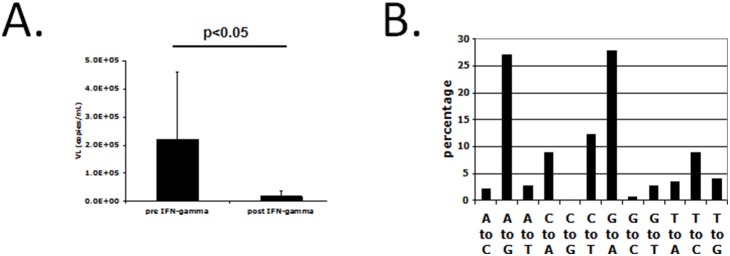
Reduction of lung HIV-1 RNA levels and HIV-1 envelope mutations associated with aerosol IFN-γ treatment. (**A**) HIV-1 RNA levels in bronchoalveolar lavage (BAL) fluids of 5 HIV-1/*M. tb* co-infected patients before (pre IFN-γ) and after (post IFN-γ) treatment with aerosolized IFN-γ. HIV-1 RNA viral load at post-treatment was significantly reduced in BAL fluids (p<0.05; mean±SE). (**B**) Nucleotide mutations around the V3 region of HIV-1 envelope post IFN-γ treatment. The percentages of mutations found in the V3 region were calculated by comparing virus sequences before and after IFN-γ therapy. A to G and G to A mutation occurred significantly as compared with other mutations (p<0.01).

We conducted a clinical trial with aerosolized IFN-γ as an adjuvant treatment for patients with and without multi-drug resistant tuberculosis [25], [26], [27], [28]. Five hundred µg/day (approximately 10 million units/day) of IFN-γ was delivered to the lung space three times a week for four weeks [29]. Five HIV-1/TB patients were saline-lavaged just after starting anti-tuberculosis treatment and after competing one month of adjunctive aerosol IFN-γ treatment. Lung HIV-1 RNA levels were significantly reduced at the end of the IFN-γ treatment period (2.2±2.4×10^5^ copies/ml vs. 1.8±2.0×10^4^ copies/ml, (mean±SE); p<0.05 **Figure 1A**). The HIV-1 envelope gp120 V3 sequence in patients’ BAL fluid demonstrated a significant number of mutations when clones obtained before IFN treatment were compared to clones obtained after treatment in the same patient. We sequenced 10 sub-clones of the viral envelope RNA in each patient and a significant number (27%: p<0.01) of these mutations post IFN-γ therapy were A to G transitions. In addition, a large proportion of mutations were G to A transitions (**Figure 1B**). In contrast, no A to G transitions were observed from 10 sub-clones of viral RNA in the BAL fluid of a HIV/TB patient who received **only** conventional mycobacterial treatment (data not shown). We sequenced 10 sub-clones of the viral RNA in their plasma and found no A to G transitions in blood-derived HIV-1 after IFN-γ therapy or after conventional mycobacterial treatment, suggesting the mutational effect of aerosol IFN-γ treatment was localized to the lung. The plasma viral load (VL) was not significantly changed following IFN-γ treatment (4.7±0.9×10^6^ copies/ml vs. 5.4±0.9×10^6^ copies/ml (mean±SE); p = 0.7). These observations are compatible with previous observations that aerosol distribution is limited to the lung compartment [29].

### Hot spots of amino acid replacement around the envelope glycoprotein V3 region after IFN-γ treatment without induction of an X4-tropic virus genotype

**Figure 2:**
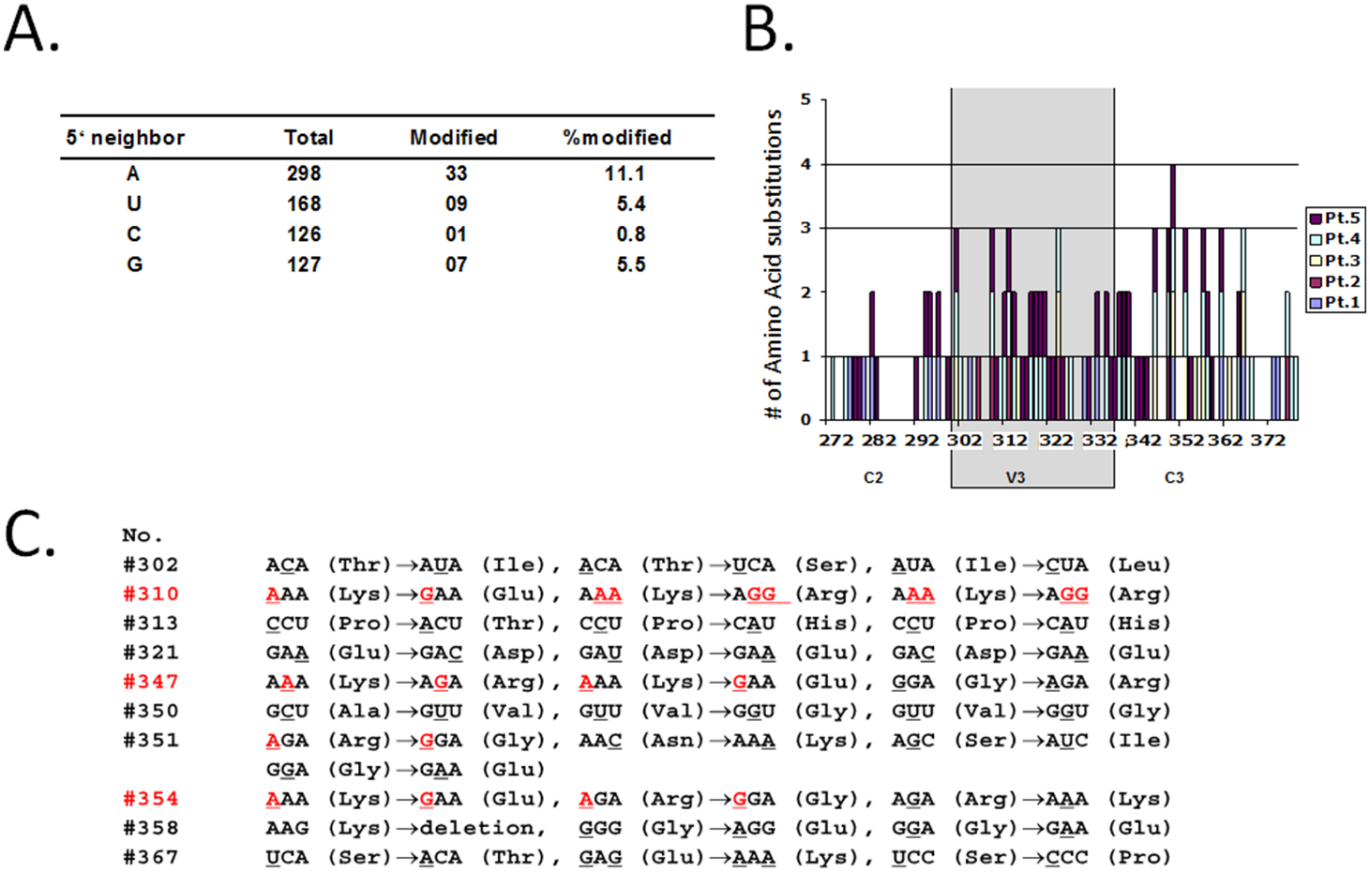
Characteristics of HIV-1 envelope mutations after IFN-γ treatment *in* *vivo*. (**A**) Frequency of modification occurring at adenosines with different 5′ nearest neighbors. Adenosine in the preferential selection of 5′ neighbors for ADAR1 (A>>U = G>C). (**B**) Numbers of amino acid substitutions around HIV-1 envelope glycoprotein V3 region. X-axis number is the amino acid position is counted from N-terminus of the envelope protein. (**C**) Amino acid substitutions resulted from by A to G mutations are shown in red.

Previous observations suggest that there is a preferential selection of adenosines for modification by ADAR1, with a 5′ neighbor preference [30]. We analyzed the sequences from paired samples of patients before and after aerosol IFN-γ treatment and noticed a similar tendency for the adenosine preferential selection of the 5′ neighborhood (A>>U = G>C) suggesting, at least in part, that mutations from A to I are caused by ADAR1 (**Figure 2A**).

**Figure 3:**
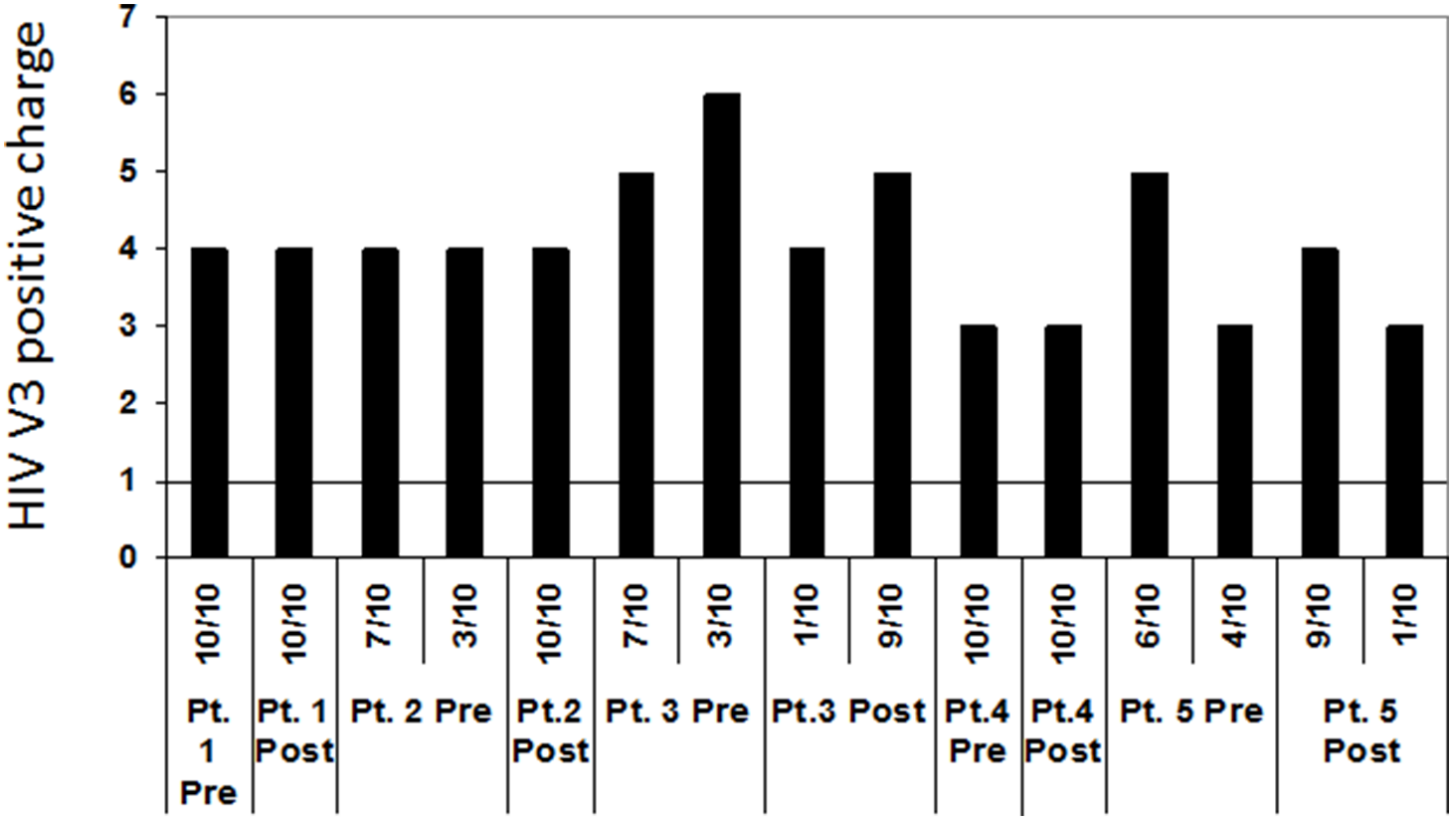
Comparison of total positive charges in the V3 loops of viruses isolated from patients at pre- and post- IFN-γ treatment. No increase in positive charges was observed in any patients after the treatment.

Due to the importance of the envelope glycoprotein V3 region in determining co-receptor usage, amino acid substitutions occurring around V3 region were compared before and after IFN-γ treatment. Sequence analysis from five paired HIV/TB co-infected patients revealed that there were 10 hot spots of amino acid replacement around the V3 region after IFN-γ treatment (**Figure 2B**). We define hot spots as sites that demonstrate amino acid substitutions seen in three or more of the five patients. Hot spots existed not only within the hyper-variable V3 region but also in the relatively conserved C2 and C3 regions. Indeed, the constant region C3 (amino acid #351) had the most frequent amino acid hot spots in this treatment group. The substitutions at amino acid #310, #347, and #354 were predominantly A to G (**Figure 2C**). These mutations did not increase the charge of the V3 region (a correlate of CXCR4 usage), but instead reduced it. Total positive charges in V3 regions are used as one marker of co-receptor preference usage [31]. We previously observed in the context of HIV/TB co-infection that there is a tendency for X4 virus to be selectively isolated in the lung space [2], [32]. *In vitro* studies also showed IFN-γ augmented susceptibility of MDM to infection with X4 virus [33], [34]. However, *in* *vivo* IFN-γ treatment did not increase the charge, suggesting IFN-γ treatment did not select X4 virus *in* *vivo* (**Figure 3**). IFN-γ treatment also did not result in substitutions to basic amino acids at positions #311 and/or #325 of the envelope protein that correlate with CXCR4 co-receptor usage (also referred to positions #11 and/or #25 positions of the V3 loop). The amino acid of these positions remained neutral or acidic after IFN-γ treatment (data not shown).

### The 150-kDa ADAR1 isoform (ADAR1L) is induced by IFN-γ or HIV-1 infection in primary macrophages but not in primary CD4 T cells

**Figure 4:**
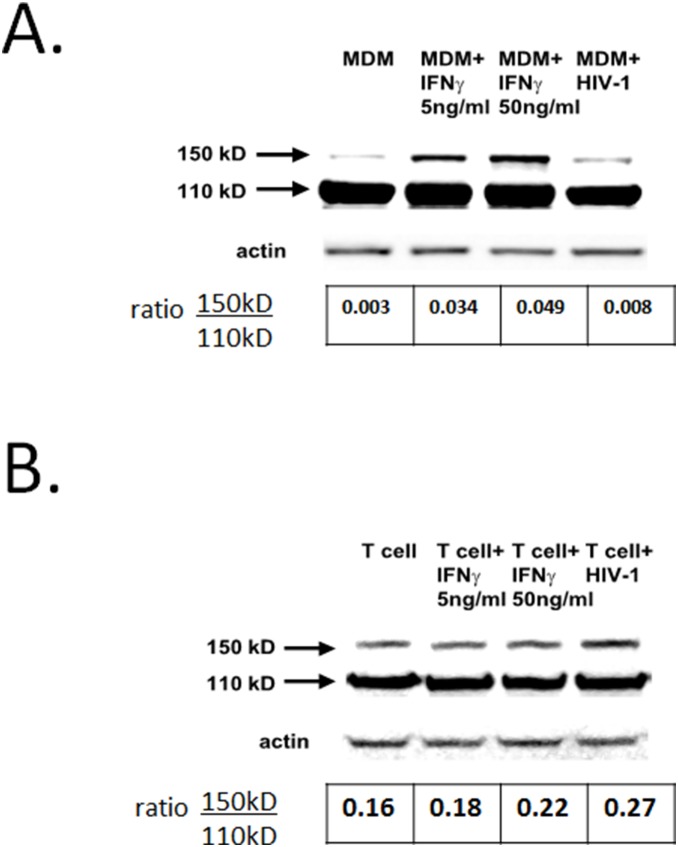
ADAR1 expression in primary macrophages and T cells *in* *vitro*. (**A**) IFN-γ induced 150-kDa ADAR1L in MDM. Cells were treated for six hours with the indicated concentrations of IFN-γ or infected with HIV-1 overnight. Immune-blot of actin was used as loading control. The ratio 150-kDa (IFN inducible) ADAR1L isoform to 110-kDa (constitutive) ADAR1S is shown below each lane. (**B**) IFN-γ did not induce ADAR1L in primary CD4+ T cells. CD4+ T cells were incubated with IFN-γ or HIV-1 as in panel (**A**).

Since ADAR1 is induced by IFNs in several cell types [16], we examined by Western blot whether IFN-γ induced ADAR1 in macrophages. Prior to antiretroviral therapy stimulation or infection, monocyte-derived macrophages (MDM) strongly express the 110-kDa (constitutive) form, whereas the 150-kDa isoform was weakly expressed, the ratio of the long to short form being 0.003. Six hours after the addition of various concentrations (0.5–500 ng/ml) of IFN-γ, the 150-kDa (IFN-inducible) ADAR1L isoform was induced, and 50 ng/ml of IFN-γ increased to the ratio to 0.049 (**Figure 4A** and data not shown). ADAR1L induction was observed even after overnight incubation with IFN-γ (data not shown). We also investigated the regulation of ADAR1L in primary CD4 T cells. In the absence of stimulation the ratio of ADAR1L to ADAR1S was 0.16, higher than observed in stimulated MDM (**Figure 4B**). However, there was little change in ADAR1L or ADAR1S with IFN-γ treatment or HIV-1 infection, suggesting different mechanisms of ADAR1 induction in macrophages and T cells.

### ADAR1 expression in the lung

**Figure 5:**
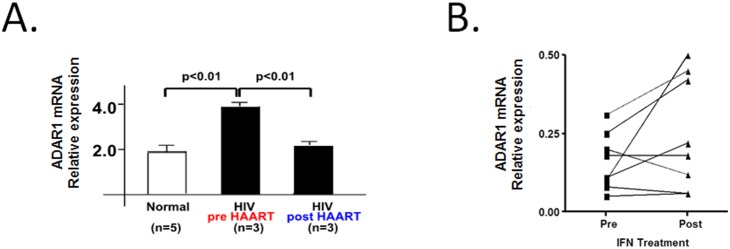
ADAR1 mRNA expression in the human lung. (**A**) ADAR1 mRNA levels were higher in HIV-1-infected patients before anti-retroviral treatment and reduced to normal after treatment (p<0.01, mean±SE). ADAR1 mRNA expression was measured by high-density cDNA arrays. Before anti-retroviral therapy and after 4 weeks of antiretroviral therapy in BAL cells of the same patients. Normal indicates mRNA from BAL cells of uninfected volunteers. (**B**) ADAR1 mRNA expression was measured by high-density cDNA arrays in 8 patients before and one-month after treatments with aerosolized IFN-γ. IFN-γ treatment was associated with about 2 fold increase in of ADAR1 mRNA (0.15±0.03 vs 0.25±0.07; mean±SE).

We evaluated the effect of antiretroviral therapy *in* *vivo* on the expression of ADAR1 mRNA using high-density cDNA arrays by analyzing mRNA of BAL cells from three HIV-1 infected individuals before versus one month after antiretroviral therapy, as well as from five normal volunteers. Antiretroviral therapy significantly reduced plasma VL in all three individuals [24]. In pre-antiretroviral therapy specimens, ADAR1 mRNA was higher as compared to normal volunteers, suggesting ADAR1 was induced in response to HIV-1 replication. However, a post-antiretroviral therapy, the levels of ADAR1 mRNA returned to those of normal volunteers (**Figure 5A**). These HIV-1 patients and volunteers did not have any lung diseases and their BAL cells consisted of more than 90% alveolar macrophages. We then examined the ADAR1 mRNA expression in BAL cells of 8 TB patients treated with IFN-γ. Half had an increase in ADAR1 mRNA expression while only one had a decrease (**Figure 5B**).

### siRNA knock-down of ADAR1 increases HIV-1 replication in primary macrophages

**Figure 6:**
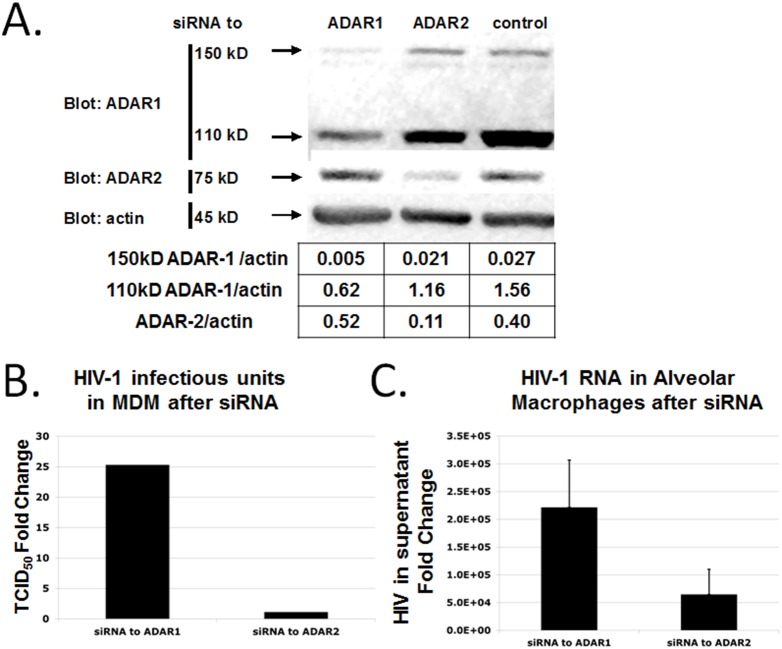
HIV-1 replication following ADAR1 siRNA knockdown in MDM infected *in* *vitro* and alveolar macrophages from HIV-1 seropositive patients. (**A**) Knockdown of ADAR1 (L+S) by siRNA in MDM. siRNA for ADAR1 led to attenuated expression of both isoforms while siRNA specific to ADAR2 did not attenuate expression of either isoform as compared to negative control. (**B**) Induction of HIV-1 replication in MDM after ADAR1 siRNA treatment. 50% tissue culture infectious dose (TCID_50_) infectivity assay was used to measure virus infectivity in the supernatant of siRNA treated MDM. TCID_50_ assay was performed 5 days after HIV-1 infection. Infectivity was normalized to that of control. No induction of virus replication was seen in cells treated with ADAR2 siRNA. (**C**) Induction of HIV-1 replication by ADAR1 siRNA in alveolar macrophages of four HIV-infected patients on antiretroviral therapy. Two days after the knock down of ADAR1 gene with siRNA, the amount of virus in the culture supernatant was significantly increased as compared to ADAR2 siRNA control (p<0.05).

To test the effect of ADAR1 expression in primary macrophages we used an siRNA knockdown strategy. We transfected MDM with ADAR1 siRNA specific to both isoforms. Transfection with ADAR1 siRNA but not ADAR2 or scrambled siRNA reduced expression of both isoforms of ADAR1 as revealed by Western blotting (**Figure 6A**). Two days after siRNA transfection, HIV-1 was added to the MDM cells for 5 days. Supernatants were harvested, and *de* *novo* virus content determined by 50% the tissue culture infectious dose (TCID50) (**Figure 6B**). Infectivity was significantly increased when ADAR1 was knocked down, whereas the infectivity was un-changed when ADAR2 was knocked down. Similar results were obtained with real time PCR assays using the same supernatants (data not shown). We repeated siRNA experiments using alveolar macrophages from patients on antiretroviral therapy [24]. Plasma VLs of all patients were under the detection limit. Supernatants were harvested and virus content measured by quantitative RT-PCR (**Figure 6C**). Again, virus replication was significantly increased when ADAR1 was knocked down (p<0.05).

### Replication of HIV-1 induces ADAR1 expression *in* *vitro*

**Figure 7:**
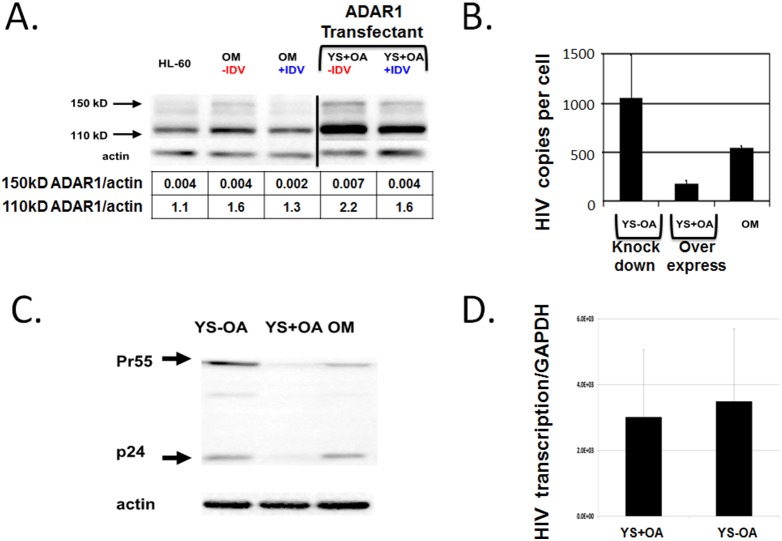
ADAR1 overexpression and knock down in a chronically HIV-1 infected macrophage cell model system. (**A**). Chronic HIV-1 infection induced ADAR1 *in* *vitro*. Expression of 150 kD and 110 kD ADAR1 was measured in OM10.1 cells and YS+OA cells treated with and without indinavir (IDV). OM10.1 is a latently HIV-1 infected macrophage cell line (HL-60 cell is the parental uninfected cell line). YS+OA cells are OM10.1 cells that stably over-express ADAR1. YS+OA cells have increased expression of the ADAR1 150 kDa isoform. Densitometry data are shown below the lanes. (**B**) HIV-1 RNA copies in the culture supernatant from ADAR1-overexpressing YS+OA were significantly lower than those of ADAR1-knocked down YS-OA. (p<0.05; mean±SE, n = 6). YS-OA is OM10.1 cells that are stably transfected with shRNA to knock down of ADAR1. (**C**) Intra-cellular p24 gag and its precursor Pr55 protein in ADAR1-overexpressing YS+OA as compared to ADAR1-knocked down YS-OA and untreated OM10.1. (**D**) Nuclear run-on transcription assays of YS+OA cells and YS-OA cells. Synthesis of HIV-1 nascent RNA was calculated as the ratio of HIV-1 RNA to GAPDH RNA.

We then developed an *in* *vitro* model to test the mechanism of ADAR1 action in macrophages. We choose the transformed HL-60 cell line latently infected with HIV-1 (OM10.1) to model chronic infection of alveolar macrophages since these cells are a major source of viral replication during OI [32], [35]. The OM10.1 cells constitutively release infectious HIV-1 virions and virus production can be inhibited by treatment with anti-HIV-1 drugs such as indinavir sulfate (IDV) [36]. We added a high concentration of IDV in the culture media for several weeks. More 150-kDa ADAR1L expression was seen in OM10.1 without IDV as compared to the parental HL-60 cell lines, while less ADAR1L was seen in OM10.1 treated with IDV (see HL-60 vs. OM-IDV vs. OM+IDV in **Figure 7A** **lanes 1 to 3**).

### ADAR1 inhibits virus replication and induces mutations similar to those observed *in* *vivo*

We hypothesized that ADAR1 may be induced by IFN-γ and the induction of ADAR1 would in turn cause mutations in HIV-1 RNA that inhibits HIV-1 replication. To test this hypothesis, we stably transfected the ADAR1 gene into OM10.1 cells to generate the cell line YS+OA. As shown in **Figure 7A** (lane 2 vs. lane 4), YS+OA expressed significantly more ADAR1L than the parental OM10.1. With IDV treatment, ADAR1L expression was decreased over several days (**Figure 7A** **, lane 4 vs. lane 5**).

We also stably transfected a plasmid expressing shRNA to the ADAR1 gene into OM10.1 cells to generate the cell line YS-OA. We confirmed the ADAR1L expression was significantly reduced in YS-OA cells (data not shown). We measured HIV-1 concentration in the supernatant from the culture of YS+OA and YS-OA cells treated with IDV to prevent the re-infection. HIV RNA from YS+OA was significantly lower (p<0.05) than that from the YS-OA (**Figure 7B**), as were gag Pr55 and p24 proteins (**Figure 7C**). However, nuclear run-on transcription rate assays show the nascent viral RNA synthesis was similar in both cell lines (n = 4, **Figure 7D**, p = 0.9). These data suggest that ADAR1 inhibits HIV-1 replication post-transcriptionally.

**Figure 8:**
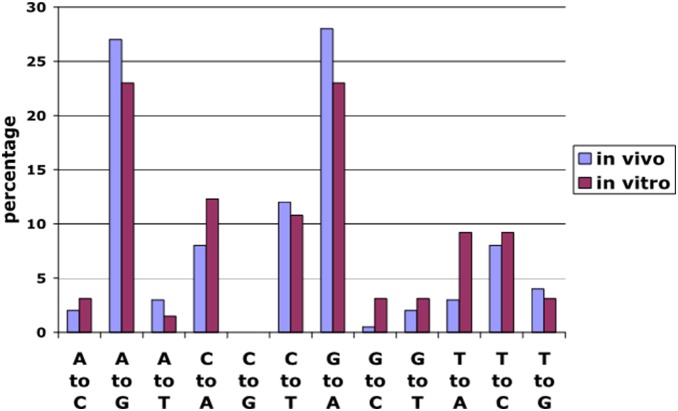
Comparison of mutations around the V3 region of HIV envelope in viruses isolated *in* *vivo* (Fig. 1B) versus mutations in viruses produced *in* *vitro* from the ADAR1-overexpressing chronically HIV-infected YS+OA cell line.

Finally, we infected MDM with replication defective VSV-NL-luc and three days after the infection added an ADAR1 expression vector to the MDM. We repeated the sequence analysis described above. The analysis of the viruses produced by MDM transfected with ADAR1 showed mutations identical to those found *in* *vivo* (**Figure 8**). These data suggest that ADAR1 may have induces mutation in the HIV-1 RNA.

As previously noted, we found a significant number of A to G mutations around the V3 region of HIV isolates from patients treated with aerosol IFN-γ. To compare these *in* *vivo* data to *in* *vitro* effects, we also sequenced the same region from viruses generated in the YS+OA cell line. There were no mutations observed in the viruses from culture supernatants when we sub-cloned the RT-PCR amplicons and sequenced more than 20 clones (data not shown). A similar result was observed when we used RNA extracts from whole cells. However, after immuno-precipitation of whole cells with anti-ADAR1 antibody and sequencing of RNA extract from the precipitate, we found the mutation pattern that was very similar to our *in* *vivo* observation (**Figure 8**), suggesting that induced ADAR1 may interact with viral RNA in the cells. When we performed immuno-precipitation experiments with anti-ADAR2 antibody, we did not find such mutations.

## Discussion

Our studies support the hypothesis that the ADAR1 gene product inhibits HIV-1 replication in human macrophages and likely produces the high frequency of A to G mutation found in HIV-1/TB co-infected patients after aerosol IFN-γ treatment. Using *in* *vivo* data from HIV-1 infected patients before and after antiretroviral therapy, we found that ADAR1 mRNA was elevated in BAL pre-antiretroviral therapy and returned to normal post-antiretroviral therapy suggesting that ADAR1 is induced during active viral replication. The actions of ADAR1 could be a double edged sword, providing an innate immune pathway that inhibits viral replication but also producing mutations that could lead to drug resistance and increased virulence.

We focused on the 150-kDa isoform because it is inducible by type I and type II IFN and is the only ADAR family member that is present in the cytoplasm [16], and, thus may mediate the post transcriptional effects. Transfection with the 150-kDa isoform produces the inhibitory effect of ADAR1 on HIV-1 replication [17]. This paper extends the observations of others into humans, in particular primary macrophages from HIV-1 positive patients and demonstrates that exogenously administered IFN-γ produces the types of alteration in ADAR1 levels and viral sequences predicted by the *in* *vitro* models.

We also demonstrate significant differences in the regulation of ADAR1 in primary lymphocytes and macrophages. Unstimulated T cells have relatively robust expression of the 150 kDa isoform as compared to MDM but interferon does not further increase the level of ADAR1 in lymphocytes. In contrast, untreated primary macrophages weakly express the 150-kDa isoform, and IFN-γ induces more than a 10 fold increase in these cells. HIV RNA has structured regions, such as TAR that bind the double-stranded RNA-binding domains of ADAR1 and PKR, another antiviral interferon inducible protein. In lymphocytes, ADAR1 participates in a multi-protein complex that inhibits the antiviral effect of PKR [37]. Some of the reported inconsistency about the activity of ADAR1 on HIV-1 replication may be due to cell type specific effects of ADAR1 [17], [19]. Here, we studied ADAR1 inhibition of HIV-1 replication in alveolar macrophages, since these are long lived cells that representing one of the potential cellular reservoirs for latent HIV infection [24].

When we knocked down ADAR1 in MDM and alveolar macrophages of patients with endogenous HIV-1 infection, the viral concentration significantly increased, suggesting that ADAR1 suppresses HIV-1 replication in these macrophages. In our transformed macrophage model, altering ADAR1 levels did not change HIV-1 transcription rate as measured by nuclear run-on assay, indicating that ADAR1 regulates HIV-1 replication post-transcriptionally.

The cell culture model produced the same pattern of ADAR1-associated mutations as observed *in* *vivo*. This was also apparent when we evaluated the effect of antiretroviral therapy. These data support the validity of this *in* *vitro* model system for investigating the anti-HIV mechanisms of ADAR1. Among the HIV-1 RNA populations present in the cell, the population specifically bound to ADAR1 was edited while those not bound to ADAR1 were not mutated. We also did not find mutations in the virions released into the culture supernatant, suggesting that the edited RNA may not be suitable for virus assembly. The existence of ribonuclease specific for inosine-containing RNA has been described, leading to the possibility that edited HIV-1 RNA is rapidly destroyed [38]. These observations may account for the absence of the mutated RNA in the cells or culture supernatants. *In vivo*, however, there must be an escape mechanism for edited HIV-1 RNA since A to G mutations are frequent.

**Figure 9:**
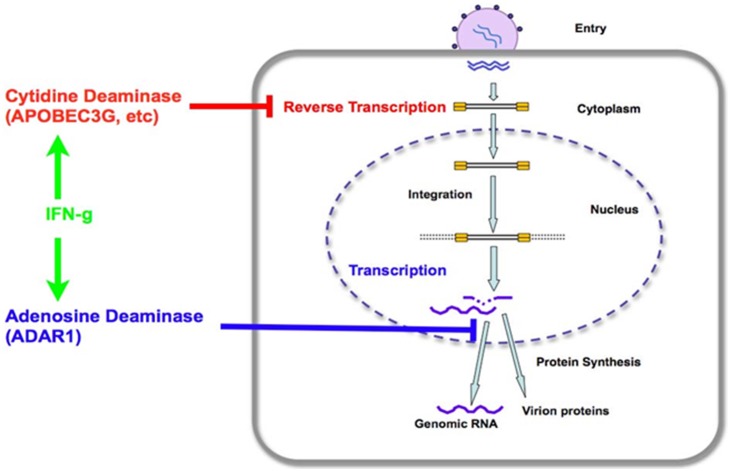
A diagram showing the distinct targets of adenosine and cytidine deaminase in the HIV-1 life cycle. IFN-γ induces both adenosine deaminase (ADAR1L) and cytidine deaminase (APOBEC3G). Adenosine deaminase may inhibit the step after viral transcription whereas cytidine deaminase acts on reverse transcription immediately after virus entry.

Our *in* *vivo* observation suggests that there might be at least two deaminases induced by aerosol IFN-γ treatment, including adenosine and cytidine deaminases. Similar to ADAR1, some cytidine deaminases are induced by IFN [39]. However, whereas the cytidine deaminase inhibits virus infection at the reverse transcription step, adenosine deaminase may affect the HIV-1replication at a post-transcription stage (**Figure 9**). Our *in* *vitro* observations suggest that there is a reciprocal expression between ADAR1 and APOBEC3G and that both molecules might contribute to anti-HIV-1 immunity. Adenosine and cytidine deaminases inhibit replication of a large number of viruses [10], [11], [13], [18] and ADAR genes are conserved in vertebrates but present only in a few invertebrates [15]. RNA editing may be one of principal mechanisms for inhibiting viral replications and be acquired during vertebrate evolution as invertebrates lack the IFN systems.

The differences between previous clinical trials of IFN-γ in HIV-1 positive patients [9] and this clinical trial, might be due to the higher dose and also the administration method of IFN-γ. In this clinical trial, 500 µg of IFN-γ was delivered via nebulizer to the lower respiratory tract, while previous trials administered 100 µg by a subcutaneous injection [25], [26], [29]. When we compared aerosol to subcutaneous IFN-γ treatment in a randomized clinical trial in South Africa, aerosol and not subcutaneous IFN-γ had a significant effect on mycobacterial smears, clinical symptoms and inflammatory cytokines in bronchoalveolar lavage cell supernatants [28]. Subcutaneous IFN-γ injection involves CD4+ T cells and macrophages while aerosol administration mainly targets alveolar macrophages, the major source of HIV-1 replication in the lung [32], [35]. ADAR1 expression induced by IFN-γ may also be different between CD4+ T cells and macrophages. Aerosol IFN treatment has several potential advantages, including the immunomodulation therapy for pulmonary tuberculosis [25], [28] and the improvement of HIV-1 infection in the lung. Since significant percentages of HIV-1 patients world-wide are co-infected with TB [1], they may benefit from the aerosol delivery approach that augments the interferon response in the lung.

## Materials and Methods

### Study population

The protocol for this study was approved by the Human Subjects Review Committees of New York University School of Medicine and Bellevue Hospital Center. All participants were provided their written informed consent to participate in this study. The ethics committees approved this consent procedure. Normal volunteers with normal chest radiographs, spirometry results, and physical examinations were recruited. HIV-1 testing was done on all patients by using enzyme-linked immunosorbent assays (ELISA), and results were confirmed by Western blotting.

#### (1) HIV-1/TB co-infected patients

Eight patients with active pulmonary tuberculosis with HIV-1 infection participated. These patients were not on antiretroviral therapy. *M. tuberculosis* isolates were recovered from sputum cultures, and 7 of 8 culture isolates were sensitive to first-line anti-tuberculosis medication. All patients had their symptoms reviewed and had chest computerized tomography scans and active evaluations relative to rIFN-γ aerosol treatment. Five patients received 500 µg of rIFN-γ (Intermune) mixed with 3 ml of normal saline via a Respigard nebulizer three times a week for 4 weeks in addition to their anti-tuberculosis medications. One patient received a conventional anti-tuberculosis medication regimen including isoniazid, rifampin, pyrazinamide, and ethambutol.

Bronchoalveolar lavage (BAL) was first performed in 8 patients 10±2 days after the start of treatment with anti-tuberculosis drugs. For one patient with multidrug-resistant tuberculosis, the first BAL was done after 12 weeks of second-line therapy. A follow-up BAL was performed approximately 1 day after the final aerosol IFN-γ treatment. For a patient who received only conventional medication, BAL was done before the treatment and after 4 weeks of the initiation of the treatment [26].

#### (2) HIV-1 patients before and after antiretroviral therapy

BAL was first performed in 3 patients before antiretroviral therapy. A follow-up BAL was done after 4 weeks of starting antiretroviral therapy [24]. BAL was performed to 4 other patients treated with antiretroviral therapy; plasma VLs of these patients were below the limit of detection. All patients had no pneumonia as confirmed by chest radiographs and standard cultures of BAL fluid. BAL cells were more than 90% alveolar macrophages.

### BAL

BAL was performed with a flexible fiber-optic bronchoscope fiber, and patients were given local anesthesia. BAL cells are representative of inflammatory and immune cells from the lung parenchyma. Normal saline (6×50-ml aliquots) was instilled and suctioned sequentially from two or three sites. The recovered fluid was filtered through sterile gauze. The total cell count was measured in a hemocytometer. Cell differentials were performed on cyto-centrifuge slides stained with Diff-Quik, and 500 cells were analyzed. Cell viability was determined by trypan blue exclusion, and in all cases, recovered cells were >90% viable.

### Reagents

All reagents were purchased from Sigma unless otherwise noted. Anti-viral compounds and all anti-HIV-1 antibodies were provided by NIH AIDS Research and Reference Reagent program.

### Purification of primary CD4+ T cells, and monocyte-derived macrophages (MDM)

Primary T cells were negatively selected from whole blood of healthy volunteers using RosetteSep CD4 T cell enrichment (StemCell Technology). For making MDM, whole blood was obtained from normal volunteers and separated into PBMCs by Ficoll-Hypaque sedimentation (Amersham Pharmacia). PBMCs were allowed to adhere to plastic plates, then harvested as monocytes. Monocytes were cultured to differentiate into MDM after 10–14 days in RPMI-1640 plus 10% bovine fetal serum.

### Cell culture

Alveolar macrophages, MDM, and primary T cells were obtained from normal volunteers with written informed consent. The protocol was approved by the IRB Committees of New York University Medical Center and Bellevue Hospital. HL-60 cells (ATCC CCL-240), Jurkat cells (ATCC TIB-152), OM10.1 (NIH AIDS Research and Reference Reagent program #1319), YS+OA (stably transfected ADAR1 gene to OM10.1 and selected with neomycin), YS-OA (stably transfected shRNA to ADAR1 gene to OM10.1 and selected with puromycin) were obtained or generated as described. All of these cells were cultured with RPMI 1640 with 10% FCS. 293T cells were provided by Dr. John S. Munger (NYU Langone Medical Center) and were cultured in DMEM with 10% FCS.

### HIV-1 and infection

Cells were infected with R5-tropic HIV-1 (BaL; NIH AIDS Research and Reference Reagent program #510) for 4 h at 37°C. HIV-1 RNA was extracted from the supernatants and assayed to determine HIV-1virus concentration. HIV-1 concentration was measured as previously described with real-time quantitative RT-PCR [32], [28]. HIV-1 infection was performed at a multiplicity of infection (MOI) of 1.0 to 10.

### HIV-1 virus infectivity

50% tissue culture infectious dose (TCID_50_) was calculated as previously described [34] according to the Reed and Muench method with modification. Instead of using phytohemagglutinin stimulated T cell blasts, we used an indicator cell line (TZM-bl from NIH AIDS Research and Reference Reagent program #8129).

### Cell extract preparation

Nuclear extracts, cytoplasmic extracts or whole cell extracts were prepared as previously described [32], [40]. Pierce BCA reagents were used to determine protein concentrations of the extracts.

### Immunoblots

Immunoblots were performed as previously described [32]. Proteins were separated by SDS-PAGE (Bio-Rad), then probed with primary antibodies (rabbit anti-ADAR1 antibody from Zymed) followed by visualization with anti-rabbit HRP antibodies (Santa Cruz) and ECL plus (Amersham).

### Transfection

The plasmid for ADAR1 expression was purchased from ATCC (MGC 45112) with the pCMV-sport6 vector. Plasmid was transfected using cationic lipids (Invitrogen) or calcium phosphate mediate (Promega) according to the manufacturer’s instructions.

To make YS+OA cells, the EcoRV-XhoI fragment containing ADAR1 cDNA was ligated into pcDNA3.1 (Invitrogen). The ADAR1 expression vector was stably transfected into OM10.1 by Gene Pulser (Bio-Rad) and was selected with 500 µg/ml of neomycin. To make the control vector, the EcoRV-NotI site containing ADAR1 cDNA was ligated into pcDNA3.1. This vector has reversely inserted ADAR1 cDNA and absence of ADAR1 protein expression was confirmed in the transfected 293T cells by Western blot.

To make YS-OA cells, a plasmid containing shRNA to ADAR1 gene was transfected into OM10.1 by Gene Pulser and was selected with 2 µg/ml of puromycin.

### siRNA and shRNA

To identify the effect of ADAR1 protein expression we used oligofectamine (Invitrogen) to transfect the annealed siRNA mixture specific to ADAR1 150-kDa and 110-kDa (top strand, 5′- CCAGCACAGCGGAGUGGUATT-3′; bottom strand, 5′-UACCACUCCGCUGUGCUGGTT-3′, from Darmacon) and specific to ADAR2 (top strand, 5′-UACAUGAGUGAUCGUGGCCUU-3′; bottom strand, 5′-GGCCACGAUCACUCAUGUAUU-3′) [41]. After 2 days cells were harvested and ADAR1 expression determined by immunoblot. A plasmid containing shRNA to knock down of ADAR1 gene was purchased from SuperArray. Oligonucleotides for shRNA to ADAR1 are 5′- CCACTTACTACGCTCCAAGAT-3′ and 5′- TGAGGGTCTTCAGCTGCATTT-3′. The negative control is 5′- GGATCTCATTCGATGCATAC-3′.

### RNA labeling and hybridization for cDNA filters

RNA labeling and hybridization for cDNA filters were performed as previously described [27], [27,40].

### Image analysis

Image analysis for cDNA filters was performed as previously described [27], [40]. Quantification of Western blot was made by ImageJ 3.1 software [42].

### Nuclear run-on transcription rate assay

The rate of viral transcription was determined by nuclear run-on with modifications described below [27], [40]. Briefly, 5×10^7^ YS+OA or YS-OA cells were collected in cell lysis buffer at 4°C. Nuclei were collected by centrifugation at 4°C. Nuclear run-on and RNA isolation were performed in the presence of biotin-16-UTP (Roche). Dynabeads M-280 were used to capture the biotin-labeled RNA molecules from the purified viral RNA, and beads were washed twice with 2x SSC plus 15% formamide and once with 2x SSC and re-suspended in RNase-free water before the preparation of random nanomer-primed cDNA. Quantitative real-time RT-PCR was performed described above. The synthesis of HIV-1 nascent RNA was calculated as the ratio of HIV-1 RNA to GAPDH RNA.

### Determination of viral sequences

The genotype of viruses was determined as described previously [2], [40]. Nested-PCR products were sub-cloned using TOPO TA Cloning kit (Invitrogen). The PCR products were sequenced by ABI PRISM autosequencer with BigDye-Deoxy Terminator Cycle Sequencing kit (Applied Biosystems).

### Virus stocks and infection

The vesicular stomatitis virus glycoprotein (VSV-G)-pseudotyped HIV-1 virus was produced as described previously [43]. Briefly, 293T cells were co-transfected with pVSV-G and pNL4-3-EGFP (NIH AIDS Research and Reference Reagent program #11100) or pNL4-3 Luc.R^−^E^−^ (NIH AIDS Research and Reference Reagent program #3418) with Lipofectamine (Invitrogen). Supernatants containing VSV-G-pseudotyped HIV-1 virus were collected 48 h after transfection. Viral supernatants were used fresh or stored at −80°C before infection. Viral concentration was measured as previously described with real-time quantitative RT-PCR as described above. Infection was performed at multiplicity of infection (MOI) at 1.0 to 10 using spinoculation (1500 g for 1 hour at room temperature). After the spinoculation, cells were washed and incubated for 48 h before flow cytometry analysis.

### Immunoprecipiation (IP) and quantitative PCR

IP experiments were performed and followed by quantitative PCR for HIV-1 as previously described [40]. Briefly cell lysates from whole cells were separated into three aliquots. The first aliquot was precipitated with anti-ADAR1 antibody or anti-ADAR2 antibody (Zymed), the second with control rabbit IgG, and the third was kept as an input. The former two samples were incubated with agarose-protein A/G beads. Captured viruses were directly extracted by TriReagent. RT-PCR was performed on the captured viruses as described above.

### Data analysis

Non-parametric data, including differences between two groups, were evaluated using the Mann-Whitney U test. To evaluate the significance of the mutation rate, the chi square goodness-of-fit test was used. A p value <0.05 was considered significant.
